# Advancing community-engaged research during the COVID-19 pandemic: Insights from a social network analysis of the trans-LINK Network

**DOI:** 10.1371/journal.pone.0271397

**Published:** 2022-11-11

**Authors:** Janice Du Mont, Nicholas Lebel, Madelaine Coelho, Joseph Friedman Burley, Sarah Daisy Kosa, Sheila Macdonald

**Affiliations:** 1 Women’s College Research Institute, Women’s College Hospital, Toronto, ON, Canada; 2 Dalla Lana School of Public Health, University of Toronto, Toronto, ON, Canada; 3 Ontario Network of Sexual Assault/Domestic Violence Treatment Centres, Toronto, ON, Canada; Sunnybrook Research Institute, CANADA

## Abstract

Collaboration across sectors is critical to address complex health problems, particularly during the current COVID-19 pandemic. We examined the ability to collaborate during the pandemic as part of a baseline evaluation of an intersectoral network of healthcare and community organizations established to improve the collective response to transgender (trans) persons who have been sexually assaulted (the trans-LINK Network). A validated social network analysis survey was sent to 119 member organizations in Ontario, Canada. Survey respondents were asked, ‘Has COVID-19 negatively affected your organization’s ability to collaborate with other organizations on the support of trans survivors of sexual assault?’ and ‘How has COVID-19 negatively affected your organization’s ability to collaborate within the trans-LINK Network?’. Data were analyzed using descriptive statistics. Seventy-eight member organizations participated in the survey (response rate = 66%). Most organizations (79%) indicated that the pandemic had affected their ability to collaborate with others in the network, citing most commonly, increased workload (77%), increased demand for services (57%), and technical and digital challenges (50%). Survey findings were shared in a stakeholder consultation with 22 representatives of 21 network member organizations. Stakeholders provided suggestions to prevent and address the challenges, barriers, and disruptions in serving trans survivors experienced during the pandemic, which were organized into themes. Seven themes were generated and used as a scaffold for the development of recommendations to advance the network, including: increase communication and knowledge exchange among member organizations through the establishment of a network discussion forum and capacity building group workshops; enhance awareness of network organizations by developing a member-facing directory of member services, their contributions, and ability to provide specific supports; strengthen capacity to provide virtual and in-person services and programs through enhanced IT support and increased opportunities for knowledge sharing and skill development; and adopt a network wide syndemic approach that addresses co-occurring epidemics (COVID-19 + racism, housing insecurity, transphobia, xenophobia) that impact trans survivors of sexual assault.

## Background

In early March 2020, the World Health Organization declared COVID-19 a pandemic and governments around the world introduced unprecedented public health restrictions to limit its transmission [1, 2]. Shelter-in-place, quarantine, and lockdown orders were imposed, limiting productivity across many sectors [2].

The impacts of COVID-19 on the research and academic sectors have been numerous and conflicting. The pandemic has sparked a massive upsurge in research outputs to guide COVID-19 public health responses [3]. However, this proliferation in pandemic-related funding streams, special journal issues, and open-access preprint databases stands in stark contrast to the significant decline in ‘non-essential’ research. For scholars whose work focuses on issues unrelated to the more formal COVID-19 response, the pandemic has significantly curtailed access to many of the resources (e.g., staff-time, access to specialized equipment, partnerships) necessary to carry out rigorous and efficient research [4].

These impacts have been particularly acute in community-engaged research (CEnR), which is predicated on “bringing transdisciplinary teams together [to] study health problems in real-world contexts” [5 p. 93]. Community-engagement in public health research and practice has long been recognized for its positive influence on a host of outcomes at the individual, community, and structural levels including, among others, enhanced accessibility of health and social services, sustained academic-community partnerships, broader dissemination and uptake of research results, and policy change [6–8].

To achieve these outcomes, literature on best practices for CEnR emphasizes the necessity of consistent, meaningful, and often in-person engagement between relevant community stakeholders and the research team (i.e., outreach, recruitment of community researchers, trust-building, collective reflection/theorizing) [5, 9–11]. However, engagement of this nature has become difficult–if not impossible–during the COVID-19 crisis, particularly for communities experiencing heightened health and social inequities during the pandemic (e.g., transgender [trans] persons who have experienced elevated risks of violence and increased social isolation and homelessness) [12]. As a result, CEnR scholars have been required to slow or radically alter their research to promote continued interaction with key stakeholders [13, 14].

### The trans-LINK Network

The trans-LINK Network is a CEnR project among researchers situated at Women’s College Research Institute, Women’s College Hospital and representatives from trans-positive community organizations and hospital-based violence treatment centres across Ontario, Canada dedicated to enhancing supports for trans survivors of sexual assault [15]. To achieve optimal levels of community engagement, the trans-LINK Network has employed a consultative CEnR approach since its inception in 2019. Taking this approach required careful consideration of a variety of contextual factors, including the scale and scope of the work [16]. Community consultative approaches emphasize the meaningful involvement of key stakeholders at critical junctures in the research process, such as in the determination of priorities and areas of focus, review of research tools, and development and promotion of recommendations based on findings [17, 18]. Consultative approaches are appropriate and feasible for use in province-wide, intersectoral projects such as the trans-LINK Network [16].

Establishment of the provincial network has also been guided by social network theory and based on a lifecycle model of network development, which outlines key activities at several sequential stages: Planning (e.g., connect key members, define purpose of network, discuss value of network), Formation (e.g., develop collaborations, negotiate network focus and identity, exchange knowledge, develop sense of collective ownership of network), Maturation (e.g., focus and expand the network), and Sustainability (e.g., continue network activities considered effective) [19].

In the planning stage of the network, we consulted with relevant stakeholders in seven community engagement meetings held across Ontario between June 7, 2019 and July 11, 2019 [20]. Representatives from 96 community and hospital-based organizations attended these meetings, with almost all (97%) in attendance expressing interest in being part of the network’s further development [20]. Principles (e.g., recognize position in the world and dialogue, acknowledge the differences between intent and impact) and activities (e.g., The World Café) informed by collaborative learning approaches were used to address power dynamics and foreground the voices of representatives [19, 21]. Key insights arising from these meetings informed the purpose of the network and five prominent areas for action, which later framed the project’s working groups: education and training, peer involvement, advocacy, accessibility, and knowledge sharing and exchange [20].

For the formation stage, a survey was undertaken early fall 2019 to examine specific involvement of member organizations in the network and further determine network values, activities, and deliverables [22]. Responding member organizations (64/93) represented a rich diversity of services including, but not limited to, counselling/mental health (77%), advocacy and outreach (67%), education and training (56%), healthcare (51%), sexual assault care (46%), and care for other forms of violence (42%) and varied in size from 100 or less employees (44%) to more than 1000 employees (16%). The input of diverse organizational representatives, of whom more than half had over a decade of experience in their role (53%) and a substantial minority were trans or gender diverse (26%) [23], was critical for further guiding the development of the network. For example, the highest prioritized deliverables–provision of standardized sensitivity training on violence against trans persons for professionals and development of an online directory/resource list of trans-affirming service providers and organizations–directly informed our next steps. After the survey, an additional 31 organizations joined the network.

At the outset of the COVID-19 pandemic, the project had just received federal funding to continue mobilizing partnerships within the trans-LINK Network and support its maturation. Funds were to be allocated toward convening the current 130 member organizations in a provincial in-person network-wide meeting. The meeting, scheduled for summer 2020, would serve as a platform for discussion on the governance of the network, including the formation of the working groups of member organizations and peer leaders representing trans communities. A symposium was to be held one year later with all 130 network member organizations across Ontario, peer leaders, and key policymakers with relevant expertise and influence in the area of anti-violence and/or other trans issues. The symposium would serve as an opportunity to discuss the progress of the network and engage government decision-makers in advancing its mission.

As both events became impossible under COVID-19 public health restrictions, first implemented March 2020 [24, 25], we had to strategize about how to maintain engagement of trans-LINK Network member organizations and collectively determine next steps. The imperative to advance our network was particularly urgent in light of the documented upsurge in rates of sexual assault and domestic violence and the unprecedented strain on healthcare and social service systems supporting survivors during the COVID-19 pandemic [26]. A decision was made to pivot the project to a series of virtual activities. We hosted a webinar to share the progress of the network to date (May 2020), recruited peer leaders, and formed and convened the working groups (Research & Evaluation, Education & Training, Advocacy & Accessibility, Knowledge Sharing & Exchange; September 2020-February 2021). The working groups then guided our activities, including: the implementation of a trans-LINK WebPortal (www.translinknetwork.com) with an interactive directory of services (March 2021) [27]; the launch of an e-learning curriculum for diverse providers on trans-affirming post sexual assault care (May 2021) [15, 28]; the development of a social media advocacy campaign to promote awareness to counter damaging attitudes, beliefs, and reactions related to sexual assault against trans people (June 2021) [29]; and the determination of research priorities in a national online survey of stakeholders (September 2021).

The current study was an entirely virtual baseline evaluation of the network, which identified member organizations’ potential expectations for collaborating within the trans-LINK Network. The evaluation also aimed to determine any challenges and barriers to the further development of the network generally and during the COVID-19 pandemic and how to address them. The findings could have important implications for public health researchers, practitioners, and policymakers working across sectors to maintain the delivery of essential healthcare and social services for marginalized groups during this and future public health emergencies.

## Methods

### Survey sample

Of the 130 trans-LINK Network member organizations, 119 providing direct healthcare and community-based services were invited to participate in the survey (S1 Table).

### Measure

We used the validated 19-question Program to Analyze, Record, and Track Networks to Enhance Relationships (PARTNER) survey for the purposes of our baseline evaluation [30]. The PARTNER survey has been successfully utilized to conduct social network analyses of organizational partnerships within other contexts [31–33].

As recommended by the developers of PARTNER [34], we made minor modifications to some existing survey items to better reflect the nature of the trans-LINK Network and added two items relevant to the potential impacts of the ongoing COVID-19 pandemic and one item on the frequency of collaboration. The final survey contained 22 close-ended items across three sections (7 organizational-level, 11 relational-level, 4 challenges and barriers to collaboration), as well as an additional open-ended question asking for further relevant information, if any. The survey varied in duration, depending on the number of collaborations described, ranging from approximately 10 minutes to an hour. The survey was hosted on the PARTNER platform, which was developed by network science experts at Visible Network Labs [35].

**Fig 1:**
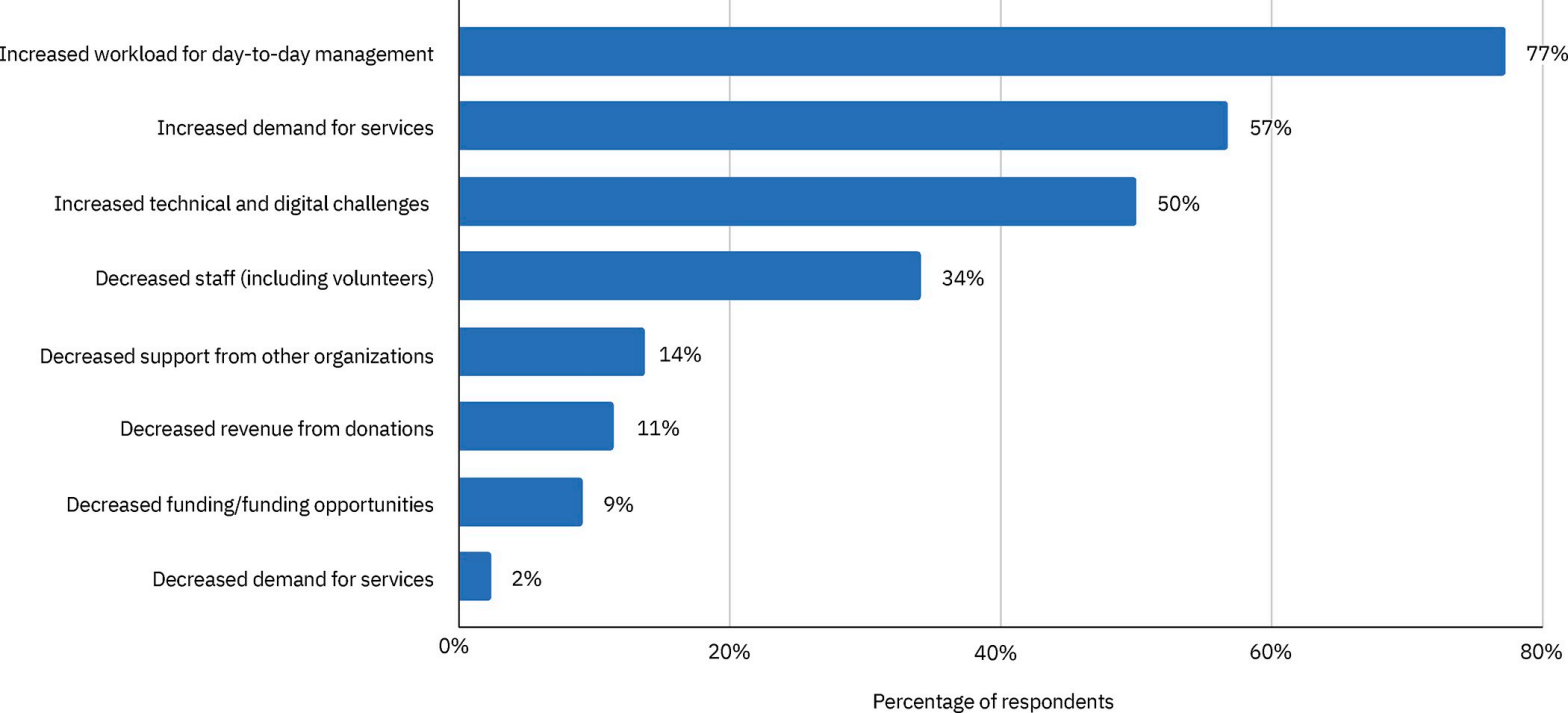
Impacts of the COVID-19 pandemic on collaboration within the trans-LINK Network (n = 44). Note: Responses are not mutually exclusive.

The current study focuses on survey items at the organizational level (Role in organization, Length of time in role, see Table 1 for list of response options; Potential contributions to the network, Aspirations for the network, Desired effects of participating in the network, and Activities necessary to achieve outcomes, see Table 2 for response options), and challenges and barriers to collaboration (Generally, what are the challenges your organization faces in collaborating with other trans-LINK Network organizations?, Has COVID-19 negatively affected your organization’s ability to collaborate with other organizations on the support of trans survivors of sexual assault?, see Table 3 for response options; and How has COVID-19 negatively affected your organization’s ability to collaborate within the trans-LINK Network?, see Fig 1 for response options). Relational-level data are described elsewhere [36].

**Table 1:** Demographics of responding representatives from trans-LINK Network member organizations.

**Role in organization**	**N = 78**	**%**
Manager or director	25	32
Coordinator	20	26
Front-line provider	20	26
Executive or board member	6	8
Educator	5	6
Support or administrative staff	1	1
Volunteer	1	1
**Length of time in role**	**N = 78**	**%**
0 to <3 Months	4	5
3 to <6 Months	0	0
6 Months to <1 Year	5	6
1 to <3 Years	23	29
3 to <5 Years	21	27
5 to <10 Years	16	21
10+ Years	9	12

**Table 2:** Organization expectations for participation in the trans-LINK Network.

**Potential contributions to the network** ^a^	**N = 72**	**%**
Advocacy and public awareness raising	52	72
Connections to organizations that are not currently members of the trans-LINK Network	49	68
Communication and media assistance (e.g., social media promotion)	34	47
Event-sharing/shared hosting opportunities	31	43
Training and professional development opportunities	26	36
Research/knowledge translation expertise and resources	18	25
Physical resources	17	24
Dedicated staff time (includes volunteers)	12	17
Strategic planning and project management	6	8
Access to funding and funding opportunities	2	3
Fiscal management assistance	1	1
Translation and interpretation supports	1	1
IT/web support (e.g., WebPortal management)	0	0
**Aspirations for the network** ^a^	**N = 67**	**%**
Improved knowledge/resource sharing	58	87
Improved availability, accessibility, and quality of services	58	87
Enhanced collaboration and connection	57	85
Reduction of healthcare disparities	54	81
Implementation of coordinated activities (e.g., training and professional development, advocacy initiatives)	52	78
Increased public awareness	50	75
Improved health and psychosocial wellbeing	48	72
Improved policies and/or guidelines	46	69
Improved referral among organizations	44	66
New sources of data	34	51
Less redundancy across regions (e.g., service provision)	27	40
**Desired effects of participating in the network** ^a^	**N = 70**	**%**
Improved accessibility/quality of programs and services	62	89
Enhanced knowledge of other services	58	83
Increased capacity for education and training	55	79
Increased capacity for advocacy	48	69
Increased capacity for prevention work	39	56
Increased referrals	38	54
Increased data sharing (informal and formal) among organizations	33	47
Increased visibility in a region or specific service area	31	44
Increased ability to translate data into action	26	37
New programs and services	26	37
Increased capacity for research and evaluation	16	23
**Activities necessary to achieve outcomes?** ^a^	**N = 67**	%
Bringing together diverse stakeholders within the network	56	84
Exchanging information	53	79
Engaging outside stakeholders (e.g., policy makers)	45	67
Having a variety of communication channels	42	63
Meeting regularly (i.e., minimum bi-monthly)	36	54
Having a shared mission and vision	36	54
Collective decision-making	34	51

^a^Responses are not mutually exclusive.

**Table 3:** Challenges and barriers to collaboration within the trans-LINK Network.

**General challenges** ^a^	**N = 69**	**%**
Lack of time to regularly participate in network activities	42	61
Not sure how to contribute	25	36
Staff turnover, retirement, and loss of institutional knowledge	24	35
Lack of capacity for advocacy work	19	28
Competing priorities of member organizations	19	28
Lack of communication within the network	19	28
Lack of funding	16	23
Distance between member organizations	11	16
Lack of leadership in the organization supporting involvement in the network	7	10
There are no challenges to participating in the network	6	9
Conflict among member organizations	1	1
**Impact of COVID-19 pandemic on collaboration**	**N = 62**	**%**
Not at all	13	21
A small amount	23	37
A fair amount	19	31
A great deal	7	11

^a^Responses are not mutually exclusive.

### Procedure

The PARTNER survey was distributed via email to the 119 trans-LINK Network member organizations providing direct service on June 22, 2021. The survey remained open for four weeks, with weekly reminder emails sent.

Each email invitation was addressed to a representative of a specific network member organization and included an individualized link to the survey, which only they could access. This link took representatives to a welcome page, which included an information sheet and consent form to complete before beginning the survey. The information sheet provided the background and purpose of the survey. The consent form specified the rights and responsibilities of participants and detailed risks, benefits, and security and confidentiality considerations. The study was approved by Women’s College Hospital Research Ethics Board in Toronto, Ontario, Canada (REB#2020-0070-E).

### Data analysis

We exported data from the PARTNER platform into Excel. We conducted descriptive analyses, including the calculation of frequencies and valid percentages for all relevant survey items. Listwise deletion was used for handling missing data (i.e., records were eliminated that had one or more missing values across variables that were specified for the analysis being conducted) [37]. Descriptions of member organizations, including type and location, were drawn from the trans-LINK Network Membership Directory (https://www.translinknetwork.com/membership-directory). Proportions were calculated for region and type of organization and compared across responding and non-responding organizations using chi-square tests.

### Stakeholder consultation

We held a virtual stakeholder consultation on zoom with trans-LINK Network member organizations in July 2021. Representatives of member organizations were invited via email to participate in the consultation, with the first 22 of 21 distinct organizations expressing interest confirmed to keep the group size small and facilitate active engagement. Participating organizations were diverse, providing sexual assault supports, 2SLGBTQI+ programming, counselling and mental health services, social and/or youth groups, healthcare, education and training opportunities, violence supports, HIV/AIDS supports, and/or advocacy and outreach.

The purpose of the consultation was to present key findings from the PARTNER survey and facilitate discussion on how to strengthen and evaluate the network moving forward. The session began with the ice breaker, Which of the findings presented stands out the most to you? Please explain why. Three questions followed: What specific action items could we adopt to enhance communication and/or connection among network member organizations?; How do we measure or evaluate the longer-term success of the network in accomplishing these action items?; and How can the network help prevent and address the challenges, barriers, and disruptions to care and support for trans survivors experienced during the COVID-19 pandemic and in future public health emergencies?

Representatives had approximately 8–10 minutes to respond to each question using the sticky note feature on Jamboard, a digital interactive whiteboard application through Google [38]. Participants were provided a link in the zoom chat from which to access Jamboard and instructions on how to use the sticky note feature. They were asked to provide as many responses to each question they had time for and to build on the responses of other members as appropriate. Responses to the questions were anonymous with no identifying information of participants visible. The consultation was facilitated by members of the research team (NL, JFB) and lasted approximately one hour. All raw data from the Jamboard activity were exported into Excel for analysis, which involved coding responses based on their content and consolidating similar codes into broader themes. For this study, only data arising from the question focused on the COVID-19 pandemic were examined. Twenty-three comments were provided by participants. From these comments, 23 distinct codes were created, which were then compiled into 7 themes.

## Results

Of the 119 network member organizations providing direct service, 78, each with one representative, participated in the survey, for a response rate of 66%. The response rate was evenly distributed across community- (66%) and hospital-based organizations (65%).

### Survey

#### Representative demographics

Most representatives from responding network member organizations were either managers or directors (32%), coordinators (26%), or front-line providers (26%) within their organization. A third (33%) of respondents reported being in their current role for 5 years or more (Table 1).

#### Organization characteristics

Approximately two-thirds of responding network member organizations were community organizations (69%) and a third were hospital-based violence treatment centres (31%). These organizations represented all regions of Ontario: Central (18%), Central West (21%), Central East (12%), East (17%), Southwest (21%), Northwest (6%), and Northeast (6%). The proportions of responding organizations by type and region were similar to those of non-responding organizations.

#### Organization expectations of participating in the network

Potential contributions to the network most commonly specified by respondents were advocacy and public awareness raising (72%), connections to organizations not currently trans-LINK Network members (68%), and communications and media assistance (47%). Least commonly specified were access to funding and funding opportunities (3%), fiscal management assistance (1%), and translation and/or interpretation supports (1%). No respondent indicated that their organization could contribute IT and/or web support to the network (Table 2).

Frequently specified aspirations for the network were improved knowledge and resource sharing (87%); improved availability, accessibility, and quality of services (87%); and enhanced collaboration and connection (85%). Common desired effects of participating in the network were improved accessibility and quality of programs and services (89%), enhanced knowledge of other services (83%), and increased capacity for education and training (79%). Most respondents indicated that bringing together diverse stakeholders within the network (84%) and/or exchanging information (79%) were necessary to achieve these outcomes (Table 2).

#### Challenges and barriers to collaboration

The most commonly cited challenges generally to collaborating within the network were a lack of time to regularly participate in associated activities (61%) and awareness of how to contribute (36%). Few respondents identified a lack of leadership supporting involvement in the network (10%) and conflict among member organizations (1%) as barriers to collaboration (Table 3). Less than one-in-ten (9%) respondents reported no challenges or barriers to participating in the network.

More than three-quarters (79%) of respondents indicated that the COVID-19 pandemic had, to some degree, affected their organization’s ability to collaborate with others in the network, with most specifying a small or fair amount (68%; Table 3). Most commonly cited impacts included: increased workload for day-to-day management (77%), increased demand for services (57%), increased technical and digital challenges (50%), and decreased staff and volunteers (34%; Fig 1). Less frequently specified impacts of the pandemic were: decreased revenue from donations (11%), reduced funding and/or funding opportunities (9%), and decreased demand for services (2%).

### Stakeholder consultation

Seven themes were identified from the stakeholder consultation data related to preventing and addressing the challenges, barriers, and disruptions to care and support for trans survivors experienced during the COVID-19 pandemic and future public health emergencies. These themes are elucidated using representative key verbatim quotations (S2 Table). The themes were then used as a scaffold for the development of recommendations to advance the trans-LINK Network (Table 4).

**Table 4:** Recommendations to advance the trans-LINK Network during the COVID-19 pandemic and future public health emergencies.

Increase communication and knowledge exchange among member organizations through the establishment of a trans-LINK Network discussion forum and capacity building group workshops
Enhance awareness of trans-LINK Network organizations by developing a member-facing directory of member services, their contributions, and ability to provide specific supports
Strengthen capacity to provide virtual and in-person services and programs through enhanced IT support and increased opportunities for knowledge sharing and skill development
Prioritize crisis supports for the mental health needs of service providers and users by expanding services to include mental health supports and focusing on harm reduction
Conduct research on trans service users’ post-COVID needs to determine future directions for programming and policy
Improve the accessibility of member organizations’ services by auditing existing policies and protocols
Adopt a syndemic approach that addresses co-occurring epidemics (COVID-19 + racism, housing insecurity, HIV, other STBBIs, transphobia, xenophobia) that impact trans survivors of sexual assault

#### Communication and knowledge exchange among member organizations

In two comments, it was indicated that, for example, it might be beneficial to host more workshops and training sessions to increase knowledge and allow for organizations with more experience in certain areas to share their expertise:

- Capacity building training and workshops for organizations/members to fill in gaps and increase knowledge.
- Workshops led by organizations that have leveraged online spaces and technology…can provide targeted recommendations to those who struggled to reach their population.

In other comments, it was suggested that member organizations could benefit from more direct communication with one another about services. One participant emphasized the desire for a way to “actively communicate service availability or changes in delivery modes with network members.” It was stressed that such communication “c[ould] also be [supported through] a chat support on the trans-LINK WebPortal, so [service providers] who do not know how to navigate or reach services [could] be supported.”

### Awareness of each other’s organizations both within and beyond the network

Three comments centered, for example, on the potential utility of developing a centralized resource within which organizations could see the “services, programs, [and] supports” of other network members. One participant highlighted the desire for the network to “develop a resource list [to]…access…and share with clients.”

#### Virtual and in-person services and programs

One participant stated a need for increased technical support to “continue to develop technology that will assist care providers in virtual care where appropriate…IT support [is] needed.” In another comment, it was suggested that the network “build a strong base not only for online support in the community but build capacity for in-person support as well, [as] many communities cannot access online supports/services.” A related need was highlighted “[to] provid[e] a variety of kinds of services and resources that are accessible on an online, self-directed, as well as [on a] person-to-person basis”.

#### Supports for mental health needs of service providers and users

One participant underscored that “support [for the] health of organization members [should include an emphasis on] burnout [and] practicing resilienc[e]”, noting a “worry for the health of organization members [which could] impact how [they] serve [their] communities.” In another comment, it was stressed, “there has been an increase in suicide rates so this is another item that should be supported (grief counselling, etc., that can be added).” It was also suggested that “safety plans [could] be used in emergency situations including harm reduction.”

#### Research and knowledge sharing on service use with trans communities

One participant noted, for instance, “we need post-COVID research for trans communities to see how their needs have changed or stayed the same (surveys, focus groups, interviews)—care may look slightly different now.”

#### Accessibility of trans-LINK Network member organizations’ services and programs

One participant asserted, “support [organizations] in reviewing policies and frameworks to make services more accessible and welcoming to trans and queer communities.” It was also noted that the network should simply “create more avenues for access.”

#### Syndemic approach

One participant highlighted the need to recognize the intersectional nature of health inequity within crisis contexts, commenting that the trans-LINK Network could “perhaps explor[e] a syndemic approach to developing supportive care strategies [for trans survivors that would address] co-occurring epidemics (COVID, Racism, Housing, HIV, other STBBIs [sexually transmitted and blood-born infections], Transphobia, Xenophobia, etc.).”

## Discussion

A CEnR approach to intersectoral network development presents opportunities to enhance health equity for underserved communities [39]. This approach, however, has been upended by the logistical, economic, and systemic challenges associated with the COVID-19 pandemic [13, 14, 26, 40, 41]. As community-engaged researchers working during this crisis to advance an intersectoral network on trans-affirming care for sexual assault survivors (the trans-LINK Network), we had to embrace substantial modifications to our activities in order to evaluate our network and maintain the engagement of its stakeholders in the determination of next steps. A pivot to virtual methods, particularly the use of zoom and Jamboard for our stakeholder consultation, proved vital in ensuring that the perspectives of our network members remained meaningfully integrated into our research. Our entirely virtual baseline evaluation of the network using PARTNER also allowed for the collection of important data and generation of strategies to further its development and longer-term sustainability. Our modified approach to evaluating and advancing a CEnR network building project in the pandemic context could inform similar efforts undertaken by researchers endeavoring to facilitate the continuity of their work and the ongoing involvement of communities and stakeholders with whom they collaborate.

Key findings from the PARTNER survey on potential contributions and desired effects of participating in the network highlight avenues through which organizational aspirations to enhance collaboration and connection within the trans-LINK Network can be achieved to improve availability and accessibility of services. Organizations indicated most commonly, for example, that their contribution to the network could be in advocacy and public awareness raising, which aligned well with one of their prominent desired effects of participation, to increase capacity for advocacy. Coordination of linkages between individual member organizations with resources and expertise in advocacy with others wishing to build this capacity could bolster the activities of the network aimed at addressing the structural and systemic barriers to care faced by trans survivors [27, 42]. Similar to the operationalization of CEnR in other studies [43], this could be achieved by convening our Advocacy & Accessibility Working Group to facilitate these connections and the sharing of relevant data (e.g., needs of survivors, gaps in services) with community organizations positioned to mobilize information on the front lines of service provision. In a second example, organizations that indicated having the capacity to facilitate connections to other organizations not currently members of the trans-LINK Network–a commonly documented potential contribution—could support the enhancement of knowledge of other services, a key desired effect of participating in the network. The availability of a ‘chat’ feature on the trans-LINK Network WebPortal could support those seeking information about potential collaborators. Such chat rooms have been used successfully in CEnR to “facilitate access to local resources” [44, p. 179].

Very few organizations had not experienced general challenges to collaborating within the trans-LINK Network. Consistent with earlier research on intersectoral collaboration, the most cited challenges were lack of time to regularly participate in network activities and uncertainty on how to contribute [45, 46]. As enhanced collaboration within networks can reduce redundancies across organizations and lighten workloads [47], network members may benefit from regular meetings aimed at forming new relationships within their regions through which to indicate their assets and needs and better share responsibilities (e.g., caseloads, programming). These regional meetings could also ensure clear and ongoing communication about specific contributions of individual member organizations to the network, a strategy successfully implemented in other health-focused networks [48, 49].

The COVID-19 pandemic significantly impacted the trans-LINK Network, with a large majority of organizations indicating that their ability to collaborate had been undermined. Common challenges listed–increased workload for day-to-day management and demand for services–could be mitigated through task-sharing and knowledge exchange among network member organizations, as well as enhanced infrastructure supports during periods of crisis [50, 51]. Organizations also commonly reported challenges associated with technical and digital capacities [50, 51], although no organization indicated IT/web support as a potential contribution they could make to the network. The network may need to prioritize the enhancement of its own IT capacity, offering, for example, centralized support for virtual activities (e.g., training on how to engage with/navigate the WebPortal) and ensuring any virtual resources are as accessible to all organizations (e.g., low bandwidth options). As noted in other studies, such technical assistance is critical for successful CEnR in online platforms [52]. Despite these challenges, the advancement of cross-sectoral and collaborative approaches during the pandemic has proven valuable in addressing emergent care gaps internationally and could facilitate continuity of supports for trans people experiencing sexual assault [53, 54].

This study has limitations. First, the validated PARTNER survey allowed for only one respondent per organization. Responses, therefore, may not represent the differing perspectives and experiences of those working within an organization. Second, the burden of the pandemic may be more significant than represented herein, as those organizations most affected by the pandemic might have been unable to complete our survey or participate in our stakeholder consultation. However, the response rate to the survey was strong (66%) and the sample represented all seven regions of Ontario and a diverse range of services. Additionally, the response rate was very similar across community- and hospital-based organizations. Third, while providing stakeholder information via Jamboard was anonymous and had the advantage of putting participants at ease, we were unable to explore the potential impact of organizational characteristics on responses. Furthermore, some member organizations may have been inadvertently limited in their ability to participate in the consultation due to a lack of reliable Internet connection, particularly those in rural/remote communities. Nonetheless, video conferencing and other web-applications have been evaluated positively for use in CEnR [55].

## Conclusion

Our findings enrich public health discourse on intersectoral networks by illuminating specific challenges and barriers to participation related to the COVID-19 pandemic and underpin policy and practice recommendations to address these. Recommendations generated from the stakeholder consultation highlight the importance of ongoing communication, awareness raising, and continuity of care during periods of crisis. They also emphasize conducting and disseminating research on the impacts of the pandemic, as well as attending to the mental health needs of both service providers and users. These recommendations will guide the trans-LINK Network’s response to novel challenges during the COVID-19 pandemic and recovery period, strengthen the management of the network, enable better provision of care and supports for trans survivors, and provide a potential foundation for responding to the needs of marginalized groups in future public health emergencies. Future research will include comparisons of network outcomes by organizational type as well as across time points (biennial evaluations). In the interim, our trans-LINK Network can serve as a model for intersectoral networks bridging hospital and community organizations in the context of healthcare inequities exacerbated from COVID-19.

## Supporting information

S1 Table Description of trans-LINK Network member organizations providing front-line services and supports invited to participate in the survey. (DOCX)

S2 Table Stakeholder consultation data and analysis. (DOCX)
