# Supplementary material for: Advancing community-engaged research during the COVID-19 pandemic: Insights from a social network analysis of the trans-LINK Network
Source: PLoS One. 2022 Nov 11;17(11):e0271397. doi: 10.1371/journal.pone.0271397 (PMC9651585; doi:10.1371/journal.pone.0271397)
Supplement: S1 Table — (DOCX) [file pone.0271397.s001.docx]

**S1 Table. Description of trans-LINK Network member organizations providing front-line services and supports invited to participate in the survey (N=119).**

| Name of organization | Type of organization | Region of Ontario | Description of services/supports |
| --- | --- | --- | --- |
| 2-Spirited People of the 1^st^ Nations | Community | Central | Offers support, referrals, and advocacy. Provides HIV/AIDS education and prevention, including distribution of male and female condoms, lubricants, Indigenous resource materials, needle exchange and harm reduction materials, same sex domestic violence information and referrals, care team and caregiver trainings, social events, and free Internet access. |
| Adelaide Resource Centre for Women | Community | Central | Offers a safe and welcoming place for homeless women and provides on-site showers, phones, and laundry. Lunch provided daily. Offers help with health care, housing, life skills, and social activities. Trans women are welcome to use the services. |
| AIDS Committee of Toronto | Community | Central | Offers anonymous, confidential counselling for individuals, couples, and partners of individuals living with HIV. Provides case management and referrals, a buddy program, support groups, and health and wellness programs. Offers several support groups and programs open to both trans women and trans men. |
| David Kelley LGBTQ+ & HIV/AIDS Counselling Services, Family Services Toronto | Community | Central | Offers short-term individual, couple, and group counselling for individuals identifying as LGBTQ on a broad range of issues, including coming out, sexuality and identity, isolation, discrimination, relationship concerns, parenting, self-esteem, violence, sexual abuse, and childhood trauma. |
| Fred Victor | Community | Central | Offers a range of programs to help persons find the support they need, including shelter, job training, counselling, food access, and health information. Hosts the THRIVE! Program, a trans-positive support group that focuses on life skills, employment, and housing support. |
| Friends of Ruby | Community | Central | Provides mental health services, social services and housing for LGBTQI2S youth (16-29) including: a drop-in space with meals, activities, support groups and community; therapeutic groups and barrier-free counselling in-person and through chat, phone, and virtual conferencing; support with gender identity or transition; help accessing housing, health care, and employment; and a transitional and emergency housing facility. |
| Hassle Free Clinic | Community | Central | Offers counselling and related medical services, including birth control, testing and treatment for STIs, and anonymous HIV testing and counselling. No OHIP required. Offers services for trans women and trans men. |
| LGBT Youth Line | Community | Central | Offers peer support for 2SLGBTQ+ youth (29 and under) across Ontario via phone, text, chat, and email. Support is provided by trained peer support volunteers, with lived experience, available Sun-Fri 4-9:30 pm. Also offer local and online resources through their website and HelpLine. |
| Lumenus Community Services | Community | Central | Provides mental health services to children, youth and families including ongoing and walk in counselling at no cost. Offers supports to 2SLGBTQ+ children, youth, and their families, including drop in, outreach services, and counselling. |
| METRAC - Action on Violence | Community | Central | Offers services and programs to women and their children, as well trans populations, focused on violence prevention. Services include safety and legal information and training, youth leadership programs, workshops, and resources. |
| Pieces to Pathways, Breakaway Addiction Services | Community | Central | Hosts a peer-led substance use support program for LGBTTQQ2SIA youth aged 16-29 years in Toronto. Offers drop-ins, case management, and group counselling for LGBTTQQ2SIA youth. Also, offers expedited access to LGBTTQQ2SIA positive primary care services. |
| Pride & Prejudice, Central Toronto Youth Services | Community | Central | Offers free individual, group, and family counselling for 2SLGBTQ+ youth aged 13-24. Also offers a parent group for the caregivers of trans youth. All services are client-centered, trans affirmative, trauma informed, and grounded in anti- oppression principles. |
| Sexual Assault/Domestic Violence Care Centre, Women's College Hospital | Sexual Assault/ Domestic Violence Treatment Centre | Central | Provides emergency medical/forensic care, follow-up and counselling to women, men, and trans individuals over the age of 14. Nurse on duty 24 hours a day, 7 days a week. On-site care provided Monday 7:30am to Friday 11:00pm. Mobile service is available to surrounding Toronto Emergency Departments 24/7. |
| Sexual Harassment and Assault Resource Exchange (SHARE) Project | Community | Central | Offers confidential legal advice to workers experiencing workplace sexual harassment, gender-based harassment in the workplace, and workplace-related sexual assault. Empowers workers with information and advice about all of their options so that they can make informed decisions about which legal steps, if any, they take to address or redress their situations. All legal services are free for any worker in Ontario, regardless of age, income, and immigration status. |
| Suspected Child Abuse & Neglect Program, Hospital for Sick Children | Sexual Assault/ Domestic Violence Treatment Centre | Central | Offers care, support, and assessment to children and teens who have been maltreated and their families. Clinic is open Monday-Friday 9:00am to 5:00pm, with an on-call team available 24/7. |
| The 519 | Community | Central | Offers a meeting place for LGBTQ communities and social and recreation programs for all ages, including a wide variety of trans-specific programs. |
| Toronto Rape Crisis Centre/ Multicultural Women Against Rape | Community | Central | Provides anti-oppressive, feminist peer support to survivors of gender-based or sexualized violence through support, education, and activism. Offers a 24-hour crisis line service, individual face-to-face counselling, court support and accompaniment, advocacy, support groups, information, and referrals. |
| Trans Health Care, Sherbourne Health | Community | Central | Offers mental health counselling (transitioning, gender identity, harassment or violence, isolation, substance abuse), support groups for trans persons and families, routine primary healthcare as well as hormone therapy, workshops, special events, links to community groups, and trans specific resources and information. |
| Women's Health in Women's Hands | Community | Central | Serves racialized women 16 years of age and older in the Toronto area. Services include primary health care, mental health supports, health promotion and education, and self-advocacy programs. Committed to an inclusive feminist, pro-choice, anti-racist, anti-oppression, and multilingual participatory framework. |
| YMCA Sprott House, YMCA Greater Toronto | Community | Central | Provides residential living accommodations for up to 25 LGBTQ2S+ individuals between the ages of 16 and 24. |
| Authentic Self Counselling Services | Community | Central East | Provides general and 2SLGBTQI-affirming counselling services through an anti-oppressive, trauma informed, feminist, sex/relationship/body positive, and harm reduction lens. Services include provision of Secondary Surgery Readiness Letters. Practice is geared mainly towards adolescents, adults, couples, and families. |
| Canadian Mental Health Association, Haliburton, Kawartha, Pine Ridge | Community | Central East | Offers programming for LGBTQ persons, with many groups supporting trans persons, including Trans Peer Outreach. Currently offers all programming via Zoom. |
| Carea Community Health Centre | Community | Central East | Provides family medical care and community-based health programs that offer an LGBT safe space and supports. Hosts Colours Youth Group, a safe and social drop-in for youth 13-20 years who identify as LGBTQQ. |
| Domestic Abuse & Sexual Assault Care Centre, Mackenzie Health | Sexual Assault/ Domestic Violence Treatment Centre | Central East | Provides sexual assault care for men, women, children, and trans individuals. Also provides care for men, women, and trans individuals who are 12 years of age or older who have experienced intimate partner violence. Services are provided 24 hours a day, 7 days a week through the Emergency Department. Follow-up nursing care and counselling are provided by appointment. An LGBT-positive space. |
| Durham Regional Domestic Violence/ Sexual Assault Care Centre, Lakeridge Health Oshawa | Sexual Assault/ Domestic Violence Treatment Centre | Central East | Provides medical and forensic care to individuals who are victims of intimate partner violence and sexual assault. Available 24/7 via the Emergency Department at Lakeridge Health Oshawa. Serves adults, youth, and children. Services include acute care, follow-up care, and counselling. Committed to providing exceptional care that is inclusive, diverse, and equitable to all patients. |
| East Qrew Group, Strides Toronto | Community | Central East | Offers a safe environment for queer and trans youth 12-24 years of age where opportunities for interactive activities, discussions, and educational workshops are available. |
| Gender Diverse & Trans Program, Gilbert Centre for Social and Support Services | Community | Central East | Serves those living with HIV and LGBTQ communities living in Simcoe County and Muskoka Regions. Provides personal support and information about transitioning, community supports, and hormone therapy. Hosts a variety of social, educational, and community activities for trans and gender diverse persons, including the ongoing Trans Q* Social Group: A discreet peer-run weekly meeting for trans men, trans women, non-binary, and gender variant folks and persons who are questioning their gender identity. |
| Kawartha Sexual Assault Centre | Community | Central East | Serves survivors of all gender expressions affected by sexual and gender-based violence, offering individual and group counselling, workshops, peer support, prevention education, and professional training from a feminist, anti-oppressive/anti-racist framework. |
| Nurse-Practitioner Led Clinic, Canadian Mental Health Association Durham | Community | Central East | Offers counselling, medical treatment, hormone therapy monitoring, and more. Additional services include, but are not limited to, health education programs, sexual health services, and mental health services. |
| Prideline Durham, Distress Centre Durham | Community | Central East | Provides emotional support, crisis intervention, and community referral information specific to the concerns and issues of the LGBTQ community in Durham Region. |
| Rainbow Space Program, CAYR Community Connections | Community | Central East | Provides an inclusive and safe space for members of the LGBTQ2S community and their allies. Offers several Rainbow Rooms for those who identify with the LGBTQ2S community as well as their allies to gather and form social supports, organize recreational activities, and access information and resources on services. |
| Regional Sexual Assault & Domestic Violence Treatment Centre, Orillia Soldiers' Memorial Hospital | Sexual Assault/ Domestic Violence Treatment Centre | Central East | Provides care for all adults, teens, and children following a recent sexual assault. Intimate partner violence and child abuse and neglect services are also offered. All services are available 24 hours a day, 7 days a week. |
| Sexual Assault/Domestic Violence Care Centre, Scarborough Health Network | Sexual Assault/ Domestic Violence Treatment Centre | Central East | Provides care for women, men, and trans individuals who are 12 years of age or older and have experienced sexual assault and/or intimate violence. Services are available 24 hours a day, 7 days a week. |
| Sexual Assault/Domestic Violence Care Program, Peterborough Regional Health Centre | Sexual Assault/ Domestic Violence Treatment Centre | Central East | Provides care for all individuals who have experienced sexual assault or who are living with domestic violence. Services are offered 24 hours a day, 7 days a week through the Emergency Department. |
| York Rainbow Support, Family Services York Region | Community | Central East | Offers counselling services for LGBTTQ individuals, including couple and family counselling. Additionally, offers a support line, consultation and training, and online counselling. Offers a Trans support/drop-in group. |
| Associated Youth Services of Peel | Community | Central West | Offers confidential, free services for youth through the Youth Beyond Barriers Program, a service for youth who identify as LGBTTIQQ2S through a support and education group, individual counselling, and social media. Individual counselling is provided for youth aged 12-17 years, identifying as LGBTTIQQ2S and requiring support. |
| Bloom Clinic, WellFort Community Health Services | Community | Central West | Provides holistic care for individuals, families, and communities as the first full community health center in the region of Peel. Offers a range of programs, services, and initiatives in the areas of community capacity building, health promotion, and primary health care. Invites community members to actively participate in a process of building individual and community health with an aim of achieving a healthier community where everyone belongs. |
| Canadian Mental Health Association, Peel Dufferin | Community | Central West | Provides a public and social space for individuals that identify as lesbian, gay, bisexual, queer, Two-Spirit, trans, asexual, questioning, etc., aged 16+. Offers a group for individuals to connect with the community and gain knowledge and support on various LGBTQ+ topics/issues. |
| Chantel’s Place, Trillium Health Partners | Sexual Assault/ Domestic Violence Treatment Centre | Central West | Serves survivors of sexual assault and domestic violence regardless of gender, age, or ethnicity. Sexual assault services are offered 24 hours a day at the Mississauga Hospital Emergency Department for adults, adolescents, and children, and domestic violence services are offered to those 16 years of age and older. |
| Compass Community Health | Community | Central West | Provides an all-encompassing suite of services that adapt to the changing needs of communities within and beyond the Hamilton area. Offers a variety of 2SLGBTQ+ programming, including AFFIRM (8-week stress coping CBT group for youth), Trans ID Clinics, and Trans Femme Peer Support Groups. |
| East Mississauga Community Health Centre | Community | Central West | Promotes family and community health. Hosts the Trans Activate Program, a weekly supportive and social drop-in space for members of trans and gender-diverse communities. |
| Embrave Agency to End Violence | Community | Central West | Works within a feminist, anti-oppressive, anti-racist, disability justice, sex worker rights, and harm reduction framework to provide shelter, counselling, and advocacy supports for women, Two-Spirit, gender queer, trans and non-binary folks experiencing any form of violence in the Region of Peel and beyond. People with or without children can access services. |
| Family Services of Peel | Community | Central West | Provides counselling to LGBTTQQIAAP individuals (e.g., coming out, dealing with discrimination and social rejection, bullying, verbal, sexual and physical abuse) and family members. |
| Hamilton Trans Health Coalition | Community | Central West | Comprises family physicians, other health care providers, and trans Hamiltonians who work together to increase the capacity of Hamilton’s primary health care system to deliver high-quality health care to Trans Hamiltonians. |
| Intersections Program, Indus Community Services | Community | Central West | Provides a safe space for newcomers to receive responsive personalized counselling support and information regarding settlement with a 2SLGTBQ+ lens, strengthening social networks, and decreasing isolation. |
| Moyo Health and Community Services | Community | Central West | Provides a growing array of health promotion, education, social and support services for people living with, affected by, and at systemic risk of HIV for communities in Mississauga, Brampton, and Caledon. 2SLGBTQ+ programming includes community interventions focused on supporting cis and trans queer men, women, and gender non-conforming folks, as well as those who identify as same gender loving. Systems level collaborative work includes a leadership role at the 2SLGBTQ+ collaborative of Peel region, capacity building of service providers, and facilitation of the virtual platform Rainbow Salad (www.rainbowsalad.ca). |
| Nina’s Place, Joseph Brant Hospital | Sexual Assault/ Domestic Violence Treatment Centre | Central West | Provides sexual assault and domestic violence services for all youth and adult clients, as well as children’s services for individuals under the age of 13 years. Open 24 hours a day, 7 days a week. |
| Positive Space Network of Halton Region | Community | Central West | Offers LGBTQ+ youth drop-in programs in the Halton Region for those aged 12-25 years. These programs allow LGBTQ+ youth to connect with peers and access resources. |
| QXposure | Community | Central West | Promotes health and wellness for diverse LGBT communities in Peel Region. A partnership between the East Mississauga Community Health Centre, Associated Youth Services of Peel, and Moyo Health and Community Services. |
| Rainbow Niagara LGBTQ+ Services, Quest Community Health Centre | Community | Central West | Provides primary health care, trans specific health care, support and care for gender independent children, assessment/referral and follow-up for gender confirming surgery, mental health support/counseling, and outreach. Also provides groups and health promotion/community capacity initiatives such as: Pride Prom; Trans/Gender Questioning Youth Group; Genderquest; community presentations to social services, schools, and medical providers; support to Gay Straight Alliances, PFLAG, and various LGBTQ groups/coalitions such as Niagara’s Senior Pride Network. |
| Reach Out Centre for Kids (ROCK) | Community | Central West | Provides mental health services to children, youth, and their families, including walk-in clinics, behavioral consultations, occupational therapy services, group therapy, trauma treatment, and other psychological services. |
| Sexual Assault & Domestic Violence Treatment Program, Headwaters Health Care Centre | Sexual Assault/ Domestic Violence Treatment Centre | Central West | Responds to needs of victims of recent sexual assault regardless of gender, with services provided 24/7 through the Emergency Department at the Orangeville site. |
| Sexual Assault & Violence Intervention Services of Halton (SAVIS) | Community | Central West | Provides free, confidential, and non-judgmental 24-hour support to all survivors of violence, including women, men, and members of the trans community (24-hour crisis support, advocacy, counselling, drop-ins, and support groups). |
| Sexual Assault Centre of Brant | Community | Central West | Offers referrals to Brantford agencies accredited as Safe Spaces, which are service providers who are knowledgeable about LGBTTTIQQ issues and can provide support and information. |
| Sexual Assault/Domestic Violence Care Centre, Hamilton Health Sciences | Sexual Assault/ Domestic Violence Treatment Centre | Central West | Provides healthcare for children, adolescents, women, trans individuals, and men who have experienced sexual assault or domestic violence. Emergency Adult Services (18+): Juravinski Hospital and Cancer Centre or Hamilton General Hospital Emergency Department. Emergency Children Services (17 and under): McMaster Children’s Hospital Emergency Department. |
| Sexual Assault/Domestic Violence Care Team, Brant Community Healthcare System | Sexual Assault/ Domestic Violence Treatment Centre | Central West | Provides care with a patient-centered, feminist, anti-racist, anti-oppression, equity-based, and intersectional approach. Provides sexual assault and domestic violence care to anyone at any time post-assault. Access to the care team is available 24/7 through the Emergency Department. |
| Sexual Assault/Domestic Violence Treatment Program, Niagara Health System | Sexual Assault/ Domestic Violence Treatment Centre | Central West | Provides care 24/7 to all individuals who have experienced recent sexual assault or intimate partner abuse. |
| The AIDS Network | Community | Central West | Offers services to the 1.5 million persons living in the communities of Hamilton, Halton, Haldimand, Norfolk, and Brant. Offers a monthly full-service sexual health clinic to diverse communities of gay, bi, queer, and other men who have sex with men, whether they are cis or trans. |
| Around the Rainbow, Family Services Ottawa | Community | East | Provides education, counselling, and support services to LGBTTQ+ communities. |
| Assault & Sexual Abuse Program, Cornwall Community Hospital | Sexual Assault/ Domestic Violence Treatment Centre | East | Provides emergency services and follow-up care for women, men, and trans persons, across the lifespan, from Cornwall or the United Counties of Stormont, Dundas and Glengarry, who have recently experienced sexual assault and/or intimate partner violence. Emergency services are offered 24 hours a day, 7 days a week through the Emergency Room. |
| Assault Response & Care Centre of Leeds & Grenville, Brockville General Hospital | Sexual Assault/ Domestic Violence Treatment Centre | East | Provides non-judgmental, compassionate, trauma-informed care to individuals who have been victims of or affected by sexual assault and/or domestic violence. Emergency services are provided through Brockville General Hospital’s Emergency Department. Follow-up services offered are counselling, support groups, nursing care, peer support, and public education. |
| Care Program for Victims of Assault, Hawkesbury General Hospital | Sexual Assault/ Domestic Violence Treatment Centre | East | Provides care to address the medical, emotional, and social needs of all individuals who have experienced sexual assault, domestic violence, and/or elder abuse. Services are provided through the Emergency Department or by booking an appointment with a nurse or social worker. |
| Domestic Violence/Sexual Assault Response Program, Quinte Health Care | Sexual Assault/  Domestic Violence Treatment Centre | East | Provides acute health care for individuals, regardless of gender identity, and their families who are impacted by sexual assault and/or domestic violence. Care team responds 24 hours a day, 7 days a week to any of QHC hospital sites in Belleville, Trenton, North Hastings, and Picton. |
| H.E.A.L.T.H. Clinic Ottawa | Community | East | Serves persons 13 years of age and older, including LGBTQ+ and diverse populations, who have experienced sexual assault, coercion, and sex or labor trafficking. Services include primary healthcare, prophylaxis and preventative care, and mental health and substance use care. |
| Kind Space | Community | East | Offers social services for the health and wellness of all LGBT2SQ persons in the Ottawa/Outaouais region. Offers information resources, referral services, and support groups. |
| Lanark County Sexual Assault/Domestic Violence Program, Perth & Smiths Falls District Hospital | Sexual Assault/ Domestic Violence Treatment Centres | East | Provides a comprehensive and multi-disciplinary response to domestic violence and sexual assault, while offering immediate crisis intervention, counseling, safety planning, follow-up, referrals, advocacy, and forensic evidence collection for all individuals who have experienced sexual assault or violence. |
| LGBTQ & Allied Youth Drop-In, Leeds, Grenville and Lanark District Health Unit | Community | East | Hosts sexual health clinics in 7 communities across Leeds, Grenville, and Lanark. Provides counselling, information, and clinic services for sexuality, birth control, and pregnancy. LGBTQ Friendly nurses on staff. |
| LGBTQ+ & Trans Health Program, Centretown Community Health Centre | Community | East | Offers meetings with counsellors to explore gender transition goals, service needs, and options as well as walk-in counselling sessions for LGBT2SQ+ individuals on Wednesday afternoons. Also offers hormone initiation (ages 17+), surgical assessments, and health system navigation for trans and gender diverse individuals in the Champlain Region. |
| Ottawa Rape Crisis Centre | Community | East | Supports and empowers women, gender-fluid, non-binary, and trans survivors. Counselling services are available for all self-identified women, gender non-conforming, and gender fluid individuals 16 years and older who are either recent or childhood survivors of all types of sexual assault. Offers a crisis line and crisis, short-term, long-term, group, and support person counselling. |
| PFLAG Renfrew County | Community | East | Hosts monthly support meetings for LGBTQI2S community members and their family members. |
| Regional Assault Program, Renfrew Victoria Hospital | Sexual Assault/ Domestic Violence Treatment Centre | East | Offers support and care for women, men, and trans persons across the lifespan, who have experienced domestic violence and/or sexual assault. On-call nurses are available 24 hours a day, 7 days a week. |
| Robbie Dean Family Counselling Centre | Community | East | Offers free walk-in clinics throughout Renfrew County, parent peer support groups, LGBTQ groups, and help with connecting to mental health services. Offers a support and social group for adults who identify as LGBTQ. Offers a non-judgmental safe space for those seeking to connect with others who share similar experiences. |
| Sexual Assault & Partner Abuse Care Program, The Ottawa Hospital | Sexual Assault/ Domestic Violence Treatment Centre | East | Provides care for all individuals 16 years of age and older who have experienced sexual assault and/or domestic violence. The on-site team is available 24 hours a day, 7 days a week. A nurse-practitioner run outpatient clinic is also available. |
| Sexual Assault/Abuse Program, Children’s Hospital of Eastern Ontario (CHEO) | Sexual Assault/ Domestic Violence Treatment Centre | East | Offers care, support, and assessment to children and teens who have been maltreated, as well as their non-offending caregiver(s). |
| Sexual Assault/Domestic Violence Program, Kingston Health Sciences Centre | Sexual Assault/ Domestic Violence Treatment Centre | East | Provides sexual assault and domestic violence care for individuals 14 years of age and older who have experienced a sexual assault or domestic assault within the last 12 days. Services are available to children under the age of 14 years, regardless of when the assault or abuse took place. Care is available 24 hours a day, 7 days a week through the Emergency Department at the Kingston General or Hotel Dieu sites, or the Lennox and Addington County General Hospital. |
| Spectrum, Quinte West Youth Centre | Community | East | Provides a safe and supportive space for LGBT youth in the community and includes discussion opportunities and activities. |
| Women’s Sexual Assault Centre of Renfrew County | Community | East | Serves women over the age of 16, and their family members or partners, who have experienced sexual violence. Offers inclusive workshops, information, referrals, and a support and crisis line. |
| Alpha Court Mental Health and Addictions Services | Community | Northeast | Provides subsidized housing and case management services to individuals with a serious mental illness and/or a drug/alcohol addiction. Provides sensitive and appropriate services to LGBT persons and culturally/linguistically diverse populations. |
| Amelia Rising Sexual Assault Support Centre | Community | Northeast | Provides free, confidential support in group or individual counselling sessions for people 12 years of age and older who have experienced sexual or gender-based violence. Also provides sexual violence prevention education and a clothing/food bank for clients. Works from feminist, intersectional, anti-racist, and anti-oppressive frameworks. |
| Noojmowin Teg Health Centre | Sexual Assault/ Domestic Violence Treatment Centre | Northeast | Provides wrap-around, confidential care for individuals ages 12 and over of all gender identities who have experienced sexual and/or domestic violence. Services include clinical care, mental health and addiction services, health and wellness services, traditional health services, and cultural supports and are available to Indigenous and non-Indigenous persons across Manitoulin Island. |
| Réseau ACCESS Network | Community | Northeast | Promotes wellness, harm reduction, and education, with a particular focus on HIV/AIDS, Hepatitis C, and other health issues. Includes both client-centered and education services focused on issues faced within the LGBT community. |
| Sexual Assault Care Centre/Partner Assault Clinic, Sault Area Hospital | Sexual Assault/ Domestic Violence Treatment Centre | Northeast | Provides sexual and partner assault care to adults, adolescents, and children of all genders. The on-call team is available 24 hours a day, 7 days a week through the Emergency Department. |
| Sexual Assault Treatment Centre, North Bay Regional Health Centre | Sexual Assault/ Domestic Violence Treatment Centre | Northeast | Provides emergency health care, psychosocial support, and follow-up care for all victims and survivors of sexual assault and/or domestic violence. Care is available 24 hours a day, 7 days a week. |
| Shkagamik-Kwe Health Centre | Community | Northeast | Provides holistic and culturally relevant health services to First Nations, Metis, and Inuit individuals in the Sudbury area. Services include a Two-Spirit circle and LGBT group. |
| TG Innerselves, Sudbury Action Centre for Youth | Community | Northeast | Strives to create more inclusive and safer communities for transgender persons across Northeastern Ontario by increasing the visibility of trans persons and understanding the challenges trans persons experience in Northeastern Ontario. Offers education programs and direct client services, such as one on one support for trans persons and their families. |
| Violence Intervention & Prevention Program, Health Sciences North | Sexual Assault/ Domestic Violence Treatment Centre | Northeast | Provides many victim support services to individuals of all genders and ages who have experience sexual assault and/or domestic violence. Emergency medical services are provided by on-call nurses who are available 24 hours a day, 7 days a week through the Emergency Department. |
| Voices of Women Sudbury Sexual Assault Centre | Community | Northeast | Provides free community-based services for sexual violence survivors of all races, classes, ages, gender identities, sexual orientations, abilities, and spiritualties. Services include counselling, safety planning, therapeutic groups, and accompaniment services. |
| Assault Care & Treatment Program, Sioux Lookout Meno Ya Win Health Centre | Sexual Assault/ Domestic Violence Treatment Centre | Northwest | Promotes a partnership of community services working together to assist all individuals who have recently been sexually assaulted or have experienced intimate partner violence. First provincially and federally funded health center in Ontario that is a center of excellence in First Nations and northern healthcare. |
| Children’s Centre Thunder Bay | Community | Northwest | Hosts The Other 10%, a support group for youth and young adults between the ages of 12 and 25 who are interested in exploring what it means–and does not mean–to be a part of the LGBTQ community. Group meetings are adult-facilitated, but youth-driven and provide a safe space for participants to meet, discuss, and educate one another. Provides information and access to other community activities and resources. |
| Elevate NWO | Community | Northwest | Provides LGBTQ-inclusive services and programs to persons living with, affected by, or at risk of HIV/AIDS and Hepatitis C. Services include an HIV clinic, Hepatitis C treatment, case management (e.g., housing, health care, mental health), and emergency financial assistance. |
| NorWest Community Health Centre | Community | Northwest | Provides services, including those for LGBT individuals, focused on healthcare, counselling, and support groups for individuals/family members. Also offers transition-related medical support and trans peer support groups. |
| Ontario Native Women’s Association | Community | Northwest | Provides programs and services to Indigenous women, girls, and Two-Spirit persons, as well as their families across Ontario, including the Aboriginal Sexual Assault Worker Program, Indigenous Anti-Human Trafficking, and other community wellness and health outreach programs. |
| Sexual Assault/Domestic Violence Care & Treatment, Dryden Regional Health Centre | Sexual Assault/ Domestic Violence Treatment Centre | Northwest | Provides emergency medical care and follow-up care to women, men, and trans persons, across the lifespan, who have recently experienced sexual assault or who are victims of domestic violence. Emergency medical services can be accessed 24 hours a day, 7 days a week through the Emergency Department. |
| Sexual Assault/Domestic Violence Treatment Centre, Thunder Bay Regional Health Science Centre | Sexual Assault/ Domestic Violence Treatment Centre | Northwest | Provides emergency medical care, psycho-social treatments, and follow-up care for all victims and survivors of sexual assault and/or domestic violence. Emergency medical services and acute SA/DV care can be accessed through the Emergency Department, which is available 24/7. Non-acute and follow-up care is available Monday-Friday in the SA/DVTC Follow-up/Outreach Office. |
| Sexual Assault/Partner Abuse/SAFEKIDS Program, Lake of the Woods District Hospital | Sexual Assault/ Domestic Violence Treatment Centre | Northwest | Provides services that respond to the physical, psychological, social, and medico-legal needs of individuals who have experienced sexual assault and/or domestic violence, regardless of gender or age. Services are available 24 hours a day, 7 days a week. |
| Sexual Health Clinic, Thunder Bay District Health Unit | Community | Northwest | Provides various services, including STI and HIV testing, emergency contraception, education, and sexual health counselling. Additionally, the clinic disseminates LGBTQ resources and houses the Sexuality and Gender Equity Working Group, which is focused on supporting the LGBT2S community. |
| 2SLGBTQ+ Connection, South East Grey Community Health Centre | Community | Southwest | Provides inclusive primary care and counselling, among many other health services. Provides referrals to additional local resources depending on persons’ interests and needs. Hosts 2SLGBTQ+ Connection, a program involving drop-in meetings for networking, conversation, and informal supports for the 2SLGBTQ+ community, family, friends, and allies. Provides a safe and inclusive space. |
| ANOVA: A Future without Violence | Community | Southwest | Provides services such as safe places, shelter, support, counselling, and resources for women, their children, and oppressed individuals who have been impacted by abuse. |
| Family Services Thames Valley | Community | Southwest | Offers counselling services and a drop-in discussion and support group for persons more than 19 years of age who identify as LGBT, Two-Spirit, queer, or who are questioning. |
| Gender Intersect | Community | Southwest | Offers a social support group for trans, non-binary, and Two-Spirit people in the Guelph and Wellington region, including monthly meetings, an online community of support, and special events throughout the year. |
| Guelph Wellington Care & Treatment Centre for Sexual Assault/Domestic Violence, Guelph General Hospital | Sexual Assault/ Domestic Violence Treatment Centre | Southwest | Provides care to all individuals who have experienced sexual assault and/or domestic violence. Services are available 24 hours a day, 7 days a week through the Emergency Department. |
| HIV/AIDS Resources and Community Health (ARCH) | Community | Southwest | Offers programs, services, education, and support to persons living with, affected by, and at risk of HIV and AIDS in Guelph, Wellington County, Dufferin, Grey, and Bruce Counties. Offers a variety of peer-led trans support services as well as a trans health clinic. |
| Intersex London | Community | Southwest | Provides a non-judgmental, compassionate approach while working to create a more accepting society for intersex individuals and their families through support, education, and advocacy. |
| London Cross Cultural Learner Centre | Community | Southwest | Offers settlement services and supports for LGBTQ immigrants settling in Canada. Provides information and orientation, problem solving, and assistance with immigration and Canadian documentation. Hosts a LGBTQ social/support group for newcomers. |
| London InterCommunity Health Centre | Community | Southwest | Offers programs and services for persons exploring their gender identity, including one-to-one support and group sessions. Hosts Gender Journeys, a support group for discussion of social, emotional, and physical aspects of gender change. Includes a trans health team offering medical and support services for individuals transitioning. |
| OK2BME, KW Counselling Services | Community | Southwest | Provides free, supportive services for LGBTQ2+ identified children, teens and their families in Waterloo Region. The OK2BME program consists of three unique areas including confidential counselling services (in person, or online), OK2BME Youth Groups for individuals 12-18, as well as public education, consulting, and training around LGBTQ2+ issues. |
| Pozitive Pathways Community Services | Community | Southwest | Provides support, education, and outreach services for persons living with, affected by, or at-risk of HIV and/or HCV. Offers several social groups in Chatham, Leamington, and Windsor for LGBTQ+ Youth. |
| Regional HIV/AIDS Connection | Community | Southwest | Provides practical support, counselling services, and programming to diverse populations of persons living with and affected by issues related to HIV/AIDS. Hosts Open Closet, a social support group for LGBT2Q+ youth between 14 and 18 years. |
| Regional Sexual Assault & Domestic Violence Treatment Centre, St. Joseph’s Health Care London | Sexual Assault/  Domestic Violence Treatment Centre | Southwest | Provides emergency and follow-up care for women, men, and trans persons, across the lifespan, who have experienced sexual assault and/or domestic violence. Nurses are available on-call 24 hours a day. |
| SafeSpace London | Community | Southwest | Hosts a crisis support center for sex workers, allies, women, and non-binary individuals. |
| Sexual Assault & Partner Abuse Centre, Grey Bruce Health Services | Sexual Assault/ Domestic Violence Treatment Centre | Southwest | Provides prompt care and resources for all individuals who have experienced sexual assault and/or partner violence. A Sexual Assault Nurse Examiner is available 24 hours a day, 7 days a week. |
| Sexual Assault Support Centre of Waterloo Region | Community | Southwest | Offers support services for survivors of sexual violence, including a 24-hour support line, counselling, support groups, public education, referrals, and a court support program. |
| Sexual Assault/Domestic Violence Treatment Centre, Bluewater Health | Sexual Assault/ Domestic Violence Treatment Centre | Southwest | Provides care for women, men, and trans persons, across the lifespan, who have experienced sexual assault and/or domestic violence. Care is available 24 hours a day, 7 days a week through the Emergency Department. |
| Sexual Assault/Domestic Violence Treatment Centre, Chatham-Kent Health Alliance | Sexual Assault/ Domestic Violence Treatment Centre | Southwest | Provides care for all individuals who have experienced assault and/or domestic violence. Care is available 24 hours a day, 7 days a week through the Emergency Department. |
| Sexual Assault/Domestic Violence Treatment Centre, Windsor Regional Hospital | Sexual Assault/ Domestic Violence Treatment Centre | Southwest | Hosts three programs that focus on sexual assault, intimate partners violence, and care for children, respectively. Services are available to all survivors regardless of age or gender. Services are available 24 hours a day, 7 days a week. |
| Trans Wellness Ontario | Community | Southwest | Offers a support group for trans persons helping trans persons as well as peer mentorship, advocacy, community referrals, service navigation, human rights assistance, counselling, and group support meetings. |
| Two Spirit Group of Windsor | Community | Southwest | Facilitates an online support group for Two-Spirit persons, their families, friends, and allies. Hosts sharing circles twice a month and a craft day every other month. |
| Waterloo Region Sexual Assault/Domestic Violence Treatment Centre, St. Mary’s General Hospital | Sexual Assault/ Domestic Violence Treatment Centre | Southwest | Responds 24/7 to the needs of individuals who have experienced sexual assault, domestic violence, and human trafficking. Offers emergency and short-term follow-up care to those accessing the team of nurses and social workers. Operates through the Emergency Departments of St. Mary’s General Hospital and Cambridge Memorial Hospital. Works in partnership with many local resources geared to help clients receive the best care and support during their experience. |
| Windsor Pride Community Education and Resource Centre | Community | Southwest | Aims to create a culture of belonging for Windsor Essex’s 2SLGBTQIA people and their families, allies, employers, and educators through education, empowerment, and support programs and services. |
