# Supplementary material for: Advancing community-engaged research during the COVID-19 pandemic: Insights from a social network analysis of the trans-LINK Network
Source: PLoS One. 2022 Nov 11;17(11):e0271397. doi: 10.1371/journal.pone.0271397 (PMC9651585; doi:10.1371/journal.pone.0271397)
Supplement: S2 Table — (DOCX) [file pone.0271397.s002.docx]

| **S2 Table. Stakeholder consultation data and analysis.** | | |
| --- | --- | --- |
|  |  |  |
| **Verbatim quotations** | **Codes assigned** | **Themes generated** |
| “Can also be a chat support on the trans-LINK WebPortal so [service providers] who do not know how to navigate or reach services can be supported.” | Establish chat support on WebPortal | Communication and knowledge exchange among member organizations |
| “Actively communicate service availability or changes in delivery modes with network members.” | Communicate changes in availability or delivery modes of services |  |
| “Sharing amongst each other what worked well and what didn't.” | Share lessons learned during COVID-19 pandemic |  |
| “Workshops led by organizations that have leveraged online spaces and technology. This way they can provide targeted recommendations to those who struggled to reach their population.” | Leverage expertise through workshops from organizations that successfully utilized online technology |  |
| “Capacity building training and workshops for organizations/members to fill in gaps and increase knowledge.” | Build capacity through training/workshops to fill gaps and increase knowledge |  |
| “Offer more from stronger connected regions to others if wanted.” | Foster inter-regional connections |  |
| “Develop a resource list so we can access services and share with clients.” | Create a resource list of services to share with clients | Awareness of network member organizations |
| “Increased knowledge for participating organizations amongst the network would help for future public health emergencies.” | Increase knowledge of member organizations |  |
| “By developing a main resources list with all the services, programs, [and] supports.” | Create a resource list of services, programs, and supports |  |
| “I think the clear listing of who can contribute how will help (i.e., potentially enhancing the directory or [having] a member facing directory so that it's easy to get those details).” | Develop listing of potential contributions by member organizations |  |
| “Getting the word out on what agencies and organizations are open and continuing to provide care.” | Promote awareness of organizations who are open and continuing to provide care |  |
| “Providing a variety of kinds of services and resources that are accessible on an online, self-directed, as well as [on a] person-to-person basis.” | Provide resources accessible online, self-directed, and on a person-to-person basis | Virtual and in-person services and programs |
| “Build a strong base not only for online support in the community but build capacity for in person support as well, many communities cannot access online supports/services.” | Build a base for online support and build capacity for in-person support |  |
| “Continue to develop technology that will assist care providers in virtual care where appropriate. Again, IT support needed” | Enhance access to IT support to assist provider in delivery of virtual care |  |
| “There has been an increase in suicide rates so this is another item that should be supported (grief counselling, etc., that can be added).” | Expand services to include more mental health supports | Supports for the mental health needs of service providers and users |
| “Safety plans that can be used in emergency situations including harm reduction.” | Develop safety plants for use with clients in emergencies (including harm reduction) |  |
| “Support the health of organization members as well - burnout, practicing resiliency, I worry for the health of organization members! That impacts how we serve our communities.” | Support the mental health of members organizations to avoid burnout and improve resilience and the services provided |  |
| “We need post-COVID research for trans communities to see how their needs have changed or stayed the same (surveys, focus groups, interviews) - care may look slightly different now.” | Conduct research on the post-COVID needs of trans survivors of sexual assault | Research and knowledge sharing on service use with trans communities |
| “Sharing research.” | Share research |  |
| “Sharing of statistics.” | Share data |  |
| “Support agencies in reviewing polices and frameworks to make services more accessible and welcoming to trans and queer communities.” | Support organizations in reviewing policies to ensure accessibility | Accessibility of member organizations' services and programs |
| “Create more avenues for access.” | Create more avenues for access |  |
| “Perhaps exploring a syndemic approach to developing supportive care strategies as a network. So addressing co-occurring epidemics (COVID, Racism, Housing, HIV, other STBBIs [sexually transmitted and blood-born infections], Transphobia, Xenophobia, etc.).” | Take a syndemic approach that addresses co-occurring epidemics (COVID + Racism, Housing, HIV, other STBBIs, Transphobia, Xenophobia, etc) | Syndemic approach |
